# Quality of life outcomes of patients with chronic rhinosinusitis after functional endoscopic sinus surgery, prospective cohort study

**DOI:** 10.1016/j.amsu.2019.03.001

**Published:** 2019-03-10

**Authors:** Rabii Laababsi, Abdulhakeem bushra, Zineb Elkrimi, Amine Allouane, Sami Rouadi, Reda Abada, Mohammed Roubal, Mohammed Mahtar

**Affiliations:** ENT Department, Face and Neck Surgery, Hospital August, 20’1953, University Hospital Centre IBN ROCHD, Casablanca, Morrocco

**Keywords:** Quality of life, Endoscopic sinus surgery, Outcomes, Chronic unilateral rhinosinusitis, Medical therapy

## Abstract

**Objectives:**

To compare the outcome of patients with unilateral CRSsNP (U CRSsNP) and bilateral CRSsNP (B CRSsNP) undergoing FESS. Also, we evaluate the impact of SNOT-22 domains to predict their quality of life (QOL) outcomes and compare these factors with those of CRSwNP group, published in previous work.

**Methods:**

A prospective cohort study was performed in the hospital 20 August,66 patients who were presented between January 2016 and December 2017 were diagnosed with CRS according to guideline recommendations, and were beforehand refractory to initial medical therapy and elected to FESS. The Sino Nasal Outcome Test-22 (SNOT-22) was used to evaluate QOL.

**Results:**

A higher significant improvement was observed between preoperative and postoperative SNOT-22 scores in U CRSsNP group [37.13 ± 9.307 versus 14.11 ± 8.531] and in B CRSsNP group [41.76 ± 6.949 versus 18.57 ± 8.495]. In the U CRSsNP group, patients having a preoperative SNOT-22 score higher than 20 points attained MCID in 88%. For the other group, patients having preoperative SNOT-22 score superior to 40 points achieved MCID in 66%. A multivariate logistic regression model found preoperative predictors that have impact on QOL outcomes.

**Conclusions:**

Outcomes from this study suggest that patients with U CRSsNP having a preoperative SNOT-22 scores between 10 and 19, and patients with B CRSsNP having a preoperative SNOT-22 scores between 10 and 19 or 20–29 had no chance of achieving an MCID improvement after FESS. Also, preoperative rhinologic symptoms and preoperative psychological dysfunction domains of SNOT-22 are helpful tools to predict improvement after FESS unlike the unilateral character of CRS.

## Highlights

• To compare the outcome of patients with unilateral and bilateral Chronic rhinosinusitis undergoing FESS (Functional endoscopic sinus surgery).
• We evaluate the impact of SNOT-22 domains to predict their quality of life outcomes.
• And compare these factors with those of group, published in previous work.

## 1 Background

Chronic rhinosinusitis (CRS) is the most common otolaryngologic disease worldwide that has a great impact on quality of life (QOL). In the United States, it affects 14–16% of the population, with annual cost of USD 4.3 billion [1,2]. In the literature, very few previous articles concerning unilateral CRS are reported. According to Ahsan and Rudralingam, CRS with or without nasal polyposis (CRSwNP; CRSsNP) is the most common cause of unilateral sinus disease in 60.3%, while knowing that unilateral sinus disease is relatively rare and represents between 2.5% and 6% [3,4]. Functional endoscopic sinus surgery (FESS) is the treatment of choice for CRS refractory to medical therapy. Because, it allows restoring ventilation and mucociliary clearance [5,6]. Several developed instruments, such as the most recent Sino Nasal Outcome Test (SNOT-22) questionnaire, has been used for quantifying changes in symptoms and predicting extent of postoperative improvement [7]. The objective of this study is to:

• Report and compare outcome of patients with unilateral CRSsNP (U CRSsNP) and bilateral CRSsNP (B CRSsNP) undergoing FESS.
• Evaluate the impact of SNOT-22 domains and especially the hypothesis of unilateral nature of CRSsNP to predict the QOL outcome after FESS.

## 2 Materials and methods

### 2.1 Study design and inclusion criteria

The participants were prospectively recruited from the tertiary care center at the department of ENT. The study included 66 patients who were classified into two groups: the first group comprises 45 patients with U CRSsNP (CRS with clinical, endoscopic and radiological signs are just unilateral) whereas the second includes 21 patients with B CRSsNP. Both of them underwent FESS, between January 2016 and December 2017, when medical treatment failed. Informed written consent was obtained in advance from all patients included in this study, which was approved by the hospital's Ethics Committee.

Medical records and patient histories were used to prospectively collect information regarding age, gender, diagnostic criteria to validate diagnosis, presence of comorbidities, and other relevant patient factors, including prior sinus surgery, absence of nasal polyps, asthma, acetylsalicylic acid intolerance (ASA) and smoking.

The diagnosis of CRS was defined by the European Position Paper on Rhinosinusitis and Nasal Polyps (EPOS) [8,9]. Prior to enrollment, all subjects had previously failed to medical management defined as a minimum of a 3 weeks course of broad spectrum antibiotics (amoxicillin 500 mg + clavulanate 125 mg twice a day), a minimum of a 3 weeks trial of topical nasal corticosteroid sprays (budesonide or fluticasone, 200 μg/day) and five days trial of systemic steroid therapy (deflazacort, 1 mg/kg of weight per day) [10].

Endoscopic surgery depended on the affected sinuses evaluated during the pre-surgical computed tomography (CT) scan. The surgical procedures were performed along the guidelines described by Messerklinger and Stammberger with modifications from Wigand [11].

It consisted of maxillary antrostomy, anterior ethmoidectomy, posterior ethmoidectomy, sphenoidotomy, frontal sinus procedures, with or without septoplasty and inferior turbinate reduction.

Post operatively, all patients were given short course of antibiotic (amoxicillin 1 g + clavulanate 125 mg) for one week. Nasal saline douching was given for one month and topical nasal corticosteroid (Fluticasone 100 mcg both nostrils once daily) was started 15 days after surgery and continued if necessary. During follow up, nasal suctioning was done, crusts were removed and nasal cavity was examined for any synechiae formation for 4 weeks.

Enrollment criteria were: patients aged 18 years or more; had CRSsNP refractory to medical therapy with preoperative CT scan of the paransal sinuses and then chosen to undergo FESS. These patients were followed for 6 months.

### 2.2 Exclusion criteria

Patients were excluded if presented CRS with nasal polyposis or benign/malignant tumor and fungal sinusitis. Also, patients with mucocele, antrochoanal polyp, chronic diseases (diabetes, tuberculosis, HIV/SIDA) or without preoperative CT scan were suspended.

### 2.3 Clinical disease severity measures

All study subjects completed a medical history, head and neck clinical examinations, sinonasal endoscopy, and CT scan as part of the standard of care.

Diagnostic nasal endoscopy was performed on each patient. The score was made by using Lund-Kennedy (LK) system [12]. In this study, no polyp formation was visible by the preoperative nasal endoscopy or during surgery.

Each study participant underwent a sinus CT scanner and was classified using the Lund–Mackay (LM) scoring system [13].

### 2.4 Quality of life questionnaire

All patients were asked to complete the SNOT-22 questionnaire 48 h before and 6 months after the surgery. It has used in clinical practice and has been proved to be the most suitable sinonasal outcome scoring system [14]. Similarly, the SNOT-22 measures 5 different underlying domains: 3 sinus-specific symptom domains (rhinologic, extranasal rhinologic and ear/facial symptoms) and 2 general health-related QOL domains (psychological and sleep dysfunction) [15].

### 2.5 Analytic strategy

Descriptive statistics were drawn up on the data, the mean was found for quantitative variables and the percentage for qualitative variables.

All the statistical analysis was carried out using statistical package SPSS-20.0. The normal distribution was assessed by using Shapiro–Wilk test and skewness kurtosis z-values.

Clinically significant improvement was defined by minimal clinically important difference (MCID). It was defined as a change of ≥½ standard deviation (SD) of the baseline SNOT-22 score. It is the minimal change in symptom or QOL after a given intervention that is perceptible and relevant to the individual patient. For SNOT-22, the MCID was ≥8.90. If treatment achieves a reduction in score of less than nine points, the patient is unlikely to perceive any real benefit [7]. The proportion of patients achieving an MCID after FESS were evaluated by categorizing patients into 10 preoperative SNOT-22 groups based on 10-point increments beginning at 10 and ending at 110.

We followed three steps for statistical analysis:

• **Step 1:** comparative study between two groups (U CRSsNP versus B CRSsNP). Wilcoxon signed-rank test was used to assess the improvement between baseline and follow up time SNOT-22 scores within each group.

Mann Whitney *U* test was used to assess the differences in preoperative and postoperative QOL outcomes between the two groups.

• **Step 2:** Evaluate preoperative predictor factors, especially the hypothesis of unilateral nature of CRS, and their impact on QOL improvement and prognosis after FESS.

Then, according to QOL after surgery, correlation and regression were used to evaluate preoperative patient factors that were associated with significant postoperative outcomes such as: history of previous sinus surgery, and the five different domains of SNOT-22. Stepwise method selection was the regression model used. A p value under 0.05 (5%) was considered statistically significant for all analyses.

The study was reported in line with the STROCSS criteria [16]. And register in open access database (UIN: researchregistry4699).

## 3 Results

### 3.1 Overall findings

Consecutive series of 76 adults diagnosed of CRSsNP were recruited. Only 66 patients completed the study whereas 10 patients were excluded because they were lost to follow-up (15%). There were 45 patients with U CRSsNP and 21 patients with B CRSsNP refractory to medical therapy and submitted to FESS.

In the U CRSsNP's group: The study group included 26 (39.4%) females and 19 (28.8%) males. The mean age range was 38.76 ± 14.17. In this study, Prior sinus surgery for CRS was the more prevalent characteristics of patients (10.6%). In the clinical history, four patients had asthma, and 3 patients are smoking. None of the patients had intolerance to aspirin.

In the B CRSsNP's group: The study group included 9 (13.6%) females and 12 (18.2%) males. The mean age range was 39.57 ± 9.96. In this study, Prior sinus surgery for CRS was the more prevalent characteristics of patients (9.1%). In the clinical history, two patients had asthma. None of the patients had intolerance to aspirin.

Demographic factors were compared between the two groups, and there were no significant differences in age (P = 0.793) and gender (P = 0.298). Similarly, there was no significant difference in medical comorbidities, including presence of asthma, smoking and prior sinus surgery.

### 3.2 Comparison of QOL improvement after FESS

A strongly statistically significant improvement was observed between the scores of pre and postoperative SNOT-22 in U CRSsNP group [37.13 ± 9.307 (IQR = 14) versus 14.11 ± 8.531 (IQR = 7)] Wilcoxon signed-rank test (z = −5.844, p < 0.05) and also in B CRSsNP group [41.76 ± 6.949 (IQR = 10) versus 18.57 ± 8.495 (IQR = 16)] Wilcoxon signed-rank test (z = −4.020, p < 0.05). In addition, a significant reduction was seen in the scores of the five different domains of SNOT-22 between the preoperative and postoperative times (Table 1).

**Table 1:** Mean change in QOL after FESS between two groups.

Group	Disease specific QOL	Preoperative (Mean ± SD)	Postoperative (Mean ± SD)	Absolute Δ (Mean ± SD)	P
**U CRSsNP**	**SNOT-22 Total**	**37.13 ± 9.307**	**14.11 ± 8.531**	**23.02 ± 8.943**	**<.005**
	Rhinologic symptoms	12.62 ± 3.413	3.67 ± 3.119	8.96 ± 3.692	<.005
	Extranasal rhinologic symptoms	6.76 ± 2.217	1.84 ± 1.665	4.91 ± 2.054	<.005
	Ear/facial symptoms	6.2 ± 2.282	2.24 ± 1.885	3.96 ± 1.953	<.005
	Psychological dysfunction	10.16 ± 3.296	4.84 ± 3.082	5.31 ± 3.383	<.005
	Sleep dysfunction	9.11 ± 2.862	4.49 ± 2.455	4.62 ± 3.172	<.005
**B CRSsNP**	**SNOT-22 Total**	**41.76 ± 6.949**	**18.57 ± 8.495**	**23.19 ± 11.134**	**<.005**
	Rhinologic symptoms	10.19 ± 2.909	4.38 ± 2.179	5.81 ± 3.371	<.005
	Extranasal rhinologic symptoms	4.33 ± 0.966	1.86 ± 1.014	2.48 ± 1.123	<.005
	Ear/facial symptoms	7.81 ± 1.99	3.43 ± 1.989	4.38 ± 2.376	<.005
	Psychological dysfunction	15.52 ± 3.219	7.24 ± 4.471	8.29 ± 5.414	<.005
	Sleep dysfunction	11.67 ± 2.652	5 ± 2.793	6.67 ± 3.568	<.005

SNOT-22: 22-item Sino-Nasal Outcome.

U CRSsNP: Unilateral Chronic rhinosinusitis without nasal polyposis.

B CRSsNP: Bilateral Chronic rhinosinusitis without nasal polyposis.

FESS: Functional endoscopic sinus surgery.

Δ: absolute change value of RSDI score.

QOL: quality of life.

SD: standard deviation.

According to the Mann-Whitney *U* test, no statistically significant difference was found in SNOT-22 score outcomes between the two groups (U CRSsNP and B CRSsNP) (p = 0.7).

On the other hand, the results demonstrated that 40 patients with U CRSsNP (88.9%) and 16 patients (76.2%) with B CRSsNP achieved the MCID improvement of 9 points after FESS. When evaluating the two groups of CRSsNP based on their preoperative SNOT-22 scores, patients with U CRSsNP and having a preoperative SNOT-22 scores between 10 and 19 had no chance of achieving an MCID improvement after FESS. As for patients with B CRSsNP, patients with preoperative SNOT-22 scores between 10-19 and 20–29 had no chance of achieving an MCID improvement after FESS (Table 2).

**Table 2:** Probability of patients with U CRSsNP and B CRSsNP achieving MCID after FESS based on preoperative SNOT-22 score.

Group	U CRSsNP n (%)	Achieving MCID (%)	B CRSsNP n (%)	Achieving MCID (%)
10–19	1	0 (0.0)	0	0 (0.0)
20–29	8	6 (75)	0	0 (0.0)
30–39	19	18 (94.7)	6	2 (33)
40–49	13	12 (92.3)	13	12 (92.3)
50–59	3	3 (100)	2	2 (100)
60–69	1	1 (100)	0	0 (0.0)

SNOT-22: 22-item Sino-Nasal Outcome.

U CRSsNP: Unilateral Chronic rhinosinusitis without nasal polyposis.

B CRSsNP: Bilateral Chronic rhinosinusitis without nasal polyposis.

FESS: Functional endoscopic sinus surgery.

MCID: Minimal clinically important difference.

### 3.3 Predictive factors influencing QOL improvement after FESS

On univariate analysis, the Spearman's rank correlation coefficient and the Mann–Whitney *U* test were used to choose predictor factors that significantly affected QOL improvement at the p ≤ 0.5 (Table 3). The multivariate logistic regression model examined 6 predictive factors that significantly affected QOL improvement. This model was able to explain 55.7% (R^2^ = 0.557) of the change in QOL. Only history of prior sinus surgery predicted less improvement in QOL after FESS. Two domains of SNOT-22: preoperative rhinologic symptoms and preoperative psychological dysfunction had a positive relationship with the absolute change value of SNOT-22 score. Preoperative psychological dysfunction of SNOT- 22 was the most important preoperative predictor. The unilateral character of CRS does not influence QOL (Table 4).

**Table 3:** Univariate analysis used to choose predictor factors that significantly affected QOL improvement (p ≤ 0.5).

Predictor factor	value of *p*
history of previous sinus surgery	0.025 *
preoperative Rhinologic symptoms	0.0001 *
preoperative Extranasal rhinologic symptoms	0.021*
preoperative Ear/facial symptoms	0.010*
preoperative Psychological dysfunction	0.0008*
preoperative Sleep dysfunction	0.020*

•: p < 0.5.

**Table 4:** Predictors of disease-specific QOL (SNOT-22) improvement after FESS (n:66).

Predictor	*B*	*p*	R^2^
Constante	−2.183	–	–
Preoperative Sleep dysfunction of SNOT-22	0.210	0.680	–
Prior sinus surgery	−11.363	0.000	–
Preoperative Rhinologic symptoms of SNOT-22	1.268	0.000	–
Preoperative Psychological of SNOT-22	0.876	0.020	0.557

SNOT-22: 22-item Sino-Nasal Outcome.

QOL: quality of life.

FESS: Functional endoscopic sinus surgery.

## 4 Discussion

In the literature, multi-institutional cohort data has demonstrated that patients with CRS (CRSwNP and CRSsNP) improve on the disease-specific quality-of-life (QOL) scores to a greater degree with surgical intervention [17]. Most of these studies compared two categories of CRS: CRSwNP and CRSsNP [18,19].

And in order to have a meaningful term to define the degree of clinically significant improvement to predict treatment outcomes, the MCID was used [7]. According to Rudmik et al., patients with CRS (CRSsNP and CRSwNP) that had a preoperative SNOT-22 score higher than 30 points receive a greater than 75% of chance of achieving an MCID [20]. In the U CRSsNP group, patients having a preoperative SNOT-22 score higher than 20 points attained MCID in 88%. In the other group, patients had a preoperative SNOT-22 score superior to 40 points achieved MCID in 66%. This finding will help to inform patients, before surgery, especially with U CRSsNP and B CRSsNP about the chances of receiving an MCID after FESS.

In the present study, the sleep dysfunction of SNOT-22 had not a positive significant impact on the QOL outcomes. Despite, recent studies have focused on the relationship between sleep dysfunction and QOL in CRS [21,22]. DeConde et al., found that the decision to undergo surgical intervention in patients with CRS is best predicted by health-related QOL domains pertaining to worse psychological impairment and sleep dysfunction [23].

FESS improves QOL across all domains of SNOT-22, essentially rhinologic symptoms which experienced the greatest profit in U CRSsNP group. But it is surprising that the rhinologic symptoms can predict a positive significant outcome after FESS in CRSsNP. According to Deconde, rhinologic symptoms were not predictive of electing surgical therapy. This finding may be explained by the selection group of patients which includes only CRSsNP, and excluding other categories of CRS.

A prior sinus surgery thought to indicate a poor prognosis after FESS. Most studies have shown the same result [24,25]. Revision surgery is considered to be more difficult because the lack of landmarks (e.g. the middle nasal concha, uncinate process, etc.) may increase the time of the surgical procedure and also increase the risk of complications [26].

It is interesting to mention firstly, unilateral character of CRS was not a significant predictor of improvement after FESS.

This study has several limitations that should be acknowledged. Firstly, the patient population with CRSsNP was obtained from a tertiary care center, making external generalizations to other surgical or nonsurgical patient populations challenging. Secondly, the sample size was small and the conclusions cannot be taken for granted. Lastly, the absence of a control group is also one of these few limitations.

However, the strengths of this study include the prospective nature of data collection, the use of a properly adapted and validated assessment instrument, the assessment of results done from the standpoint of the patient, and a follow-up of one year. Furthermore, surgeons were blinded for preoperative SNOT-22 score. The study of one category of CRS (without polyps) that compare two groups (U CRSsNP and B CRSsNP) was not yet published.

Surprisingly, there is a lack of data in the published literature regarding the outcomes of refractory unilateral CRSsNP after FESS. Most of these studies evaluated all categories of CRS: with and without polyps. The present study elucidates several important contributions by identified factors that can inform the optimal selection of patients for FESS, hence the need for the current study.

## 5 Conclusion

In conclusion, this study showed that FESS improves all domains of QOL. Outcomes from this study suggest that patients with U CRSsNP having a preoperative SNOT-22 scores between 10 and 19, and patients with B CRSsNP having a preoperative SNOT-22 scores between 10 and 19 or 20–29 had no chance of achieving an MCID improvement after FESS. Also, preoperative rhinologic symptoms and preoperative psychological dysfunction domains of SNOT-22 are two helpful tools to predict improvement after FESS in CRSsNP. However, the unilateral character of CRS is not a significant predictor of improvement after FESS.

## Ethical approval

Written informed consent for publication of their clinical details and/or clinical images was obtained from the patient.

Ethical approval has been exempted by our institution.

## Conflict of interest

All authors have no conflict of interest or financial support with this article.

## Funding sources

Funding sources This research did not receive any grant or funding from governmental or private sectors.

## Author contribution

Rabii LAABABSI: Corresponding author writing the paper.

Abdulhakeem bushra: writing the paper.

Zineb ELKRIMI: writing the paper.

Allouane Mohamed amine: writing the paper.

Reda Abada: study concept.

Sami Rouadi: study concept.

Mohamed Roubal: correction of the paper.

Mohamed Mahtar: correction of the paper.

## Conflicts of interest

The authors declare having no conflicts of interest for this article.

## Research registration number

Researchregistry4699.

## Guarantor

Laababsi rabii.

## Provenance and peer review

Not commissioned, externally peer reviewed.
